# Effect and Predictive Elements for 52 Weeks’ Telbivudine Treatment on Naïve HBeAg positive Chronic Hepatitis B

**DOI:** 10.5812/kowsar.1735143x.4203

**Published:** 2011-12-20

**Authors:** Xiao-Feng Zhu, Li-Xia Lu, Ying Wang, Kong-wen Xu, Da-jiang Li, Xia Zhu, Li Liu, Cong Liu, Jin-Rong Wang, Hong Tang, Li-Chun Wang

**Affiliations:** 1 Epidemiology Department,West China School of public Health, Sichuan University, Chengdu, China; 2 Center of Infectious Diseases,West China Hospital, Sichuan University, Chengdu, China; 3 Division of Infectious Diseases,State Key Laboratory of Biotherapy, Sichuan University, Chengdu, China

**Keywords:** Telbivudine, Hepatitis B, Chronic, Predictive Value of Tests, Alanine Transaminase, Hepatitis B Virus

## Abstract

**Background:**

Antiviral treatment with nucleoside analogs has been used for chronic hepatitis B (CHB). Each kind of nucleoside analog has its own characteristics and suitability for patients. Telbivudine (LdT, brand name: Sebivo, Beijing Novartis Pharma Ltd) is the newest nucleoside analog, with strong and rapid viral suppression. However, its resistance rate is relatively high during long-term application, due to low genetic barriers to resistance. So, it is necessary to increase the effect and reduce resistance with effective management, according to baseline factors and early on-treatment responses.

**Objectives:**

To reveal possible predictive factors of the effect of telbivudine (LdT) treatment on naïve HBeAg-positive chronic hepatitis B (CHB) patients to optimize treatment.

**Patients and Methods:**

A total 71 naïve chronic hepatitis B (CHB) patients who met the inclusion criteria were enrolled. All patients were treated with LdT 600 mg Qd for at least 52 weeks. Multiple logistic regression analyses were done to investigate the predictive values of baseline factors and responses at Week 24.

**Results:**

The reduction in hepatitis virus B (HBV) DNA level was 6.44 ± 2.38 lg copies/mL at Week 52 compared with baseline. The complete virus response (CVR), biochemical response (BR), serological response (SR), and drug resistance (DR) were 61.99%, 77.46%, 35.21%, and 8.45% respectively. By multiple regression analysis, baseline alanine aminotransferase (ALT) levels significantly affected CVR (P = 0.024, OR = 1.008), and baseline ALT and baseline HBV DNA levels were independent compact factors of SR (P = 0.012, OR = 1.007; P = 0.001, OR = 0.423). The differences in CVR, SR, and DR in patients with ALT > 120 Iu/mL compared with patients with ALT ≤ 120 Iu/mL were statistically significant. The differences in SR in patients with HBV DNA > 107 copies/mL compared with patients with HBV DNA ≤ 107 copies/mL were statistically significant. Additionally, CVR, BR, and SR were differed significantly between patients with HBV DNA lower than 300 copies/mL at Week 24 and patients with HBV DNA higher than 300 copies/mL (P = 0.000, P = 0.0016, and P = 0.000, respectively).

**Conclusions:**

There were more responders among naïve HBeAg-positive chronic hepatitis B patients with lower HBV DNA levels (especially lower than 107 copies/mL) and higher ALT values (especially higher than 120 Iu/mL at baseline) to LdT treatment. Adjustments for treatment strategy should be considered if HBV DNA > 300 copies/mL at Week 24 is observed.

## 1. Background

In recent years, much evidence has confirmed that antiviral treatment with nucleoside analogs of chronic hepatitis B (CHB) can inhibit viral replication, improve liver function, and reduce the incidence of decompensated liver diseases, including cirrhosis and liver cancer, and thus improve the prognosis of diseases. Each kind of nucleoside analog has its own characteristics and suitability for patients. So, it is necessary to find out the characteristics and impact factor of each medicine to improve its clinical application. Of the four available nucleoside analogs in China [1], telbivudine (LdT, brand name: Sebivo, Beijing Novartis Pharma Ltd) is the newest nucleoside analog, with strong and rapid viral suppression. However, its resistance rate is relatively high during its long-term application, due to low genetic barriers to resistance [2]. According to the results of the Global study, the effects can be increased and the resistance rate can be reduced with effective management according to baseline factors and early on-treatment responses [3].

## 2. Objectives

In this study, a total of 71 naïve HBeAg-positive CHB patients who received LdT monotherapy for 52 weeks were enrolled, and impact factors on effect and resistance were analyzed. We wished to identify the possible predictive factors of the effect of LdT treatment on naïve HBeAg-positive CHB patients to optimized the application of LdT.

## 3. Patients and Methods

### 3.1. Patients and Study Design

Patients who visited the outpatient clinic of West China Hospital of Sichuan University from March 2008 to August 2009 and met the inclusion criteria were enrolled in this study. The inclusion criteria were as follows: positivity for hepatitis B surface antigen (HBsAg) and hepatitis B e antigen (HbeAg) for at least 6 months and without history of antiviral therapy with nucleoside analogs; alanine aminotransferase (ALT) levels between 2 to 10 times the upper normal levels (UNL); and hepatitis virus B (HBV) DNA levels ≥ 105 copies/mL. Patients were excluded if they were coinfected with human immunodeficiency virus (HIV) or other hepatitis viruses (hepatitis virus A, hepatitis virus C, hepatitis virus E, and hepatitis virus D) or had nonalcoholic fatty liver disease (NAFLD), alcoholic liver disease, posthepatitic cirrhosis, or hepatocellular carcinoma. The diagnosis of fatty liver depended on the characteristic rise in liver enzymes, including γ-glutamyltransferase; change in ultrasound Doppler; and other underlying diseases with metabolic disorder. The diagnosis of alcoholic liver disease depended on alcohol consumption, history of alcohol use, and a special imaging examination, including ultrasound Doppler and computed tomography. Liver cirrhosis was confirmed through invasive and noninvasive techniques, including liver stiffness measurement, special imaging examination, and liver biopsy. Hepatocellular carcinoma was diagnosed by elevated A fetal protein (AFP) and characteristic imaging manifestation, including computed tomography (CT) and magnetic resonance imaging (MRI). After patients signed an informed consent form, they were given LdT 600 mg daily as antiviral treatment for at least 52 weeks. During the treatment period, patients were not given any other antiviral drugs or medicines to protect liver function.

### 3.2. Serum Assays

Analyses of liver and renal functions, which included serum levels of total bilirubin (TB), ALT, albumin (ALB), blood urea nitrogen (BUN), blood creatinine (Cr), amylase, and lipase, were performed at baseline and at Weeks 12, 24, 36, and 52 of LdT treatment using an automatic biochemistry analyzer (Olympus AU5400, Olympus Corporation, Tokyo, Japan). The status of HBsAg, HBeAg, and antibodies to HBeAg (anti-HBe) was measured by microparticle enzyme-linked immunosorbent assay (ELISA) at baseline and Weeks 24 and 52. Serum HBV DNA was quantified by Cobas TaqMan Real-Time Polymerase Chain Reaction (PCR) Assay (Roche Diagnostics) (the sensitivity level was 70 copies/mL and the maximum valid level was 109 copies/mL) at baseline and Weeks 12, 24, 36, and 52, with a linear range between 300 and 109 copies/mL. LdT-associated mutations were assessed via direct sequencing if virological breakthrough occurred during the treatment. All assays were performed in the microbiological laboratories of West China Hospital, Sichuan University.

### 3.3. Definition and Evaluation of Efficacy

Virological response (VR) was defined as the level of decline at the end of treatment. A complete virological response (CVR) was defined as a reduction in HBV DNA levels to less than the level of detection (< 300 copies/mL). Biochemical response (BR) was defined as a normalization of ALT levels. Serological response (SR) was defined as disappearance of HBeAg with or without the appearance of hepatitis B e antibody (HbeAb). Virological breakthrough was defined as an increase in serum HBV DNA > 1 log copy/mL compared to the on-treatment nadir that was confirmed in two consecutive tests. Drug resistance (DR) was defined as the emergence of virological breakthrough and the presence of drug-resistant mutations. The curative effect of LdT was assessed at Weeks 12, 24, and 52 during treatment. Rates of CVR, BR, SR, and DR were assessed at the time points above respectively. Baseline factors, including age, gender, ALT, and DNA levels, were analyzed for their effects on LdT treatment. HBV DNA levels at Week 24 were analyzed as impact factors during the treatment

### 3.4. Statistical Analysis

Quantitative data were presented as the mean ± standard deviation (SD), categorical data were presented as counts and percentages, and HBV DNA levels were presented as log-transformation. Data were analyzed using SPSS, version 13.0 (SPSS Inc., Chicago, IL). ANOVA was used to analyze the reduction in HBV DNA levels at Weeks 12, 24, and 52 respectively. Pearson chi-square was used to compare CVR, BR, SR, and DR at Week 52 between patients with different baseline factors. Logistic regression analysis was used to investigate the baseline predictors for the response to treatment. Age, baseline DNA, and baseline ALT were quantitative variables. Gender was the qualitative variable. In all cases, tests of significance were 2-tailed, and statistical significance was defined as P < 0.05.

## 4. Results

### 4.1. General Information

A total of 71 patients were included, comprising 60 (84.5%) males and 11 (15.5%) females, with ages ranging from 17 to 46 years (mean 28 ± 8.337). Baseline data were as follows: the median level of HBV DNA was 1.25 ×108 copies/mL, and the mean ALT level was 183.99 ± 131.87 IU/mL.

### 4.2. Virological Effect

**Figure 1:**
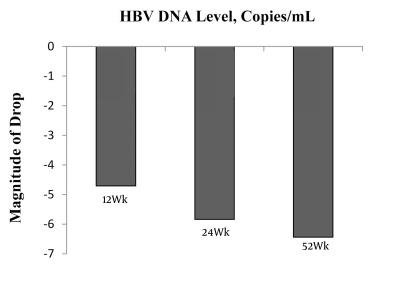
Reduction of HBV DnA Level at Weeks 12, 24 and 52 Respectively Compared With That of the Baseline

The reduction in HBV DNA levels was 4.71 ± 1.88, 5.84 ± 2.04, and 6.44 ± 2.38 lg copies/mL at Weeks 12, 24, and 52 respectively, compared with baseline (Figure 1). The difference in the magnitude of the decrease was statistically significant at the three time points (F = 12.294, P = 0.000). The prevalence of HBV DNA level < 300 copies/mL was 25.35% (18/71), 45.07% (32/71), and 61.97% (44/71) at Weeks 12, 24, and 52, respectively.

### 4.3. Biochemical Effect

ALT normalization was achieved in 39.44% (28/71), 64.79% (46/71), and 77.46% (55/71) of patients at Weeks 12, 24, and 52, respectively.

### 4.4 . Serological Effect

The disappearance of HBeAg was observed in 21.13% (15/71), 28.17% (20/71), and 35.21% (25/71) of patients at Weeks 12, 24, and 52, respectively. HBeAg/Anti-HBe seroconversion was achieved in 9.86% (7/71), 16.90% (12/71) and 23.94% (17/71) of patients at Weeks 12, 24, and 52, respectively.

### 4.5. Resistance and Side Effects

By Week 52, viral breakthrough occurred in 7 (9.86%) patients, 6 of which (8.45%) had confirmed genotypic resistance, comprising 5 cases with rtM204I and 1 case with rtLl80M + rtM204V.

Overall, LdT demonstrated a good safety profile. All levels of serum electrolytes were normal. Mild adverse reactions were observed in 4 (5.63%) patients, 3 of whom had a mild to moderate increase in blood creatine kinase without myalgia or muscle weakness at Week 24, 36, and 36, respectively. In the follow-up period, increased creatine kinase all reduced in the next 3 to 6 months without special treatment or drug withdrawal. Another patient suffered from insomnia, which disappeared after symptomatic treatment. No renal failure or pancreatitis occurred in any patient.

### 4.6. Logistic Regression Analysis of Baseline Impact Factors for Antiviral Effect of LdT

As shown in Table 1, when CVR at Week 52 was regarded as the outcome variable, the multiple logistic regression analyses of compact factors at baseline showed that only baseline ALT level (P = 0.024) was an independent compact factor of Week 52 CVR. Based on the odds ratio (OR = 1.008), it was easier to achieve undetectable HBV DNA levels at Week 52 in patients with higher ALT levels compared with patients with lower ALT levels at baseline. If BR at Week 52 was regarded as the outcome variable, the multiple logistic regression analyses of compact factors at baseline showed no impact of BR. When SR at Week 52 was regarded as the outcome variable, the multiple logistic regression analyses of compact factors at baseline showed that baseline ALT level (P = 0.012) and baseline HBV DNA level (P = 0.001) were both independent compact factors of Week 52 SR. The odds ratio (OR = 1.007 and 0.423) showed that it was easier to achieve HBeAg disappearance at Week 52 in patients with higher ALT levels and lower HBV DNA levels compared with patients with lower ALT levels and higher HBV DNA levels at baseline. When DR at week 52 was regarded as the outcome variable, the multiple logistic regression analyses of compact factors at baseline showed no impact of DR.

**Table 1:** Logistic Regression Analyses With CVR, BR, SR and DR at week 52 s outcome Variable

	**P value**	**Adjusted OR a**	**OR a (95% CI a)**
CVR^a^			
Age b	0.666	1.381	0.319–5.983
Gender c	0.075	0.420	0.153–1.062
Baseline DNA a, b	0.231	0.671	0.635–1.365
Baseline ALT a, b	0.024	1.008	1.001–1.015
BR a			
Age	0.481	0.972	0.899–1.051
Gender	0.213	0.252	0.029–2.207
Baseline DNA	0.459	0.817	0.490–2.771
Baseline ALT	0.161	0.995	0.988–1.002
SR a			
Age	0.300	0.964	0.899-1.033
Gender	0.172	2.914	0.628-13.517
Baseline DNA	0.001	0.423	0.252-0.711
Baseline ALT	0.012	1.007	1.001-1.012
DR^a^			
Age	0.589	0.965	0.848–1.098
Gender	0.119	7.237	0.600–87.286
Baseline DNA	0.104	1.000	0.999–1.001
Baseline ALT	0.230	1.008	0.981–1.005

^a^ Abbreviations: ALT, alanine aminotransferase; BR, biochemical response;CI, confidence interval; CVR, complete virus response; DnA, deoxyribose ucleic acid; DR, Drug resistance; oR, odds ratio; SR, serological esponse

^b^ Age, baseline DnA and baseline ALT are quantitative variables

^c^ Gender is qualitative variable

### 4.7 Comparison of CVR, BR, SR, and DR at Week 52 Between Patients With Baseline ALT > 120 IU/mL and ≤ 120 IU/mL

**Figure 2:**
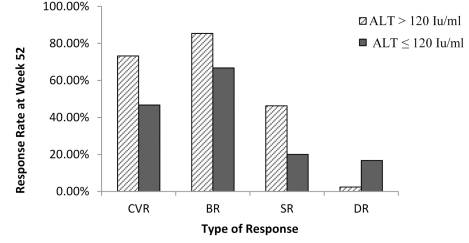
Comparison of CVR, BR, SR and DR at Week 52 Between patients ith Baseline ALT > 120IU/mL and Those With ≤ 120 IU/mL. CVR, complete virological response; BR, biochemical response; SR, serological response; DR, durg resistance

All patients were divided into two groups: baseline ALT > 120 Iu/mL and ≤ 120 Iu/mL. At Week 52, the CVR, BR, SR, and DR of 41 patients with baseline ALT > 120 Iu/mL were 73.2% (30/41), 85.4% (35/41), 46.3% (19/41), and 2.4% (1/41), respectively. In the 30 patients with baseline ALT ≤ 120 Iu/mL, the CVR, BR, SR, and DR were 46.7% (14/30), 66.7% (20/30), 20.0% (6/30), and 16.7% (5/30), respectively. The differences in CVR, SR, and DR between the two groups were statistically significant (X2 = 5.164, P =0.023; X2 = 5.269, P = 0.022; X2 = 4.533, P = 0.033). No significant difference was found in BR between the two groups (X2 = 3.470, P = 0.062) (Figure 2).

### 4.8. Comparison of CVR, BR, SR, and DR Between Patients With Baseline HBV DNA > 107 copies/mL and HBV DNA ≤ 107 copies/mL

**Figure 3:**
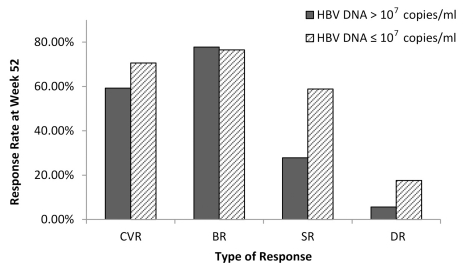
Comparison of CVR, BR, SR and DR at Week 52 Between patients With aseline HBV DnA > 107 Copies/mL and Those With HBV DnA ≤ 10 7 Copies/mL. CVR, complete virological response; BR, biochemical response; SR, serological response; DR, durg resistance

All patients were divided into two groups: baseline HBV DNA > 107 copies/mL and ≤ 107 copies/mL. At Week 52, the CVR, BR, SR, and DR of 54 patients with baseline HBV DNA > 107 copies/mL were 59.3% (32/54), 77.8% (42/54), 27.8% (15/54), and 5.6% (3/54), respectively. In the 17 patients with HBV DNA ≤ 107 copies/ml, the CVR, BR, SR, and DR were 70.6% (12/17), 76.5% (13/17), 58.8% (10/17), and 17.6% (3/17), respectively. The differences in SR between the two groups were statistically significant (X2 = 5.463, P = 0.019). No significant difference was found in CVR, BR, or DR between the two groups (X2 = 0.704, P=0.401; X2 = 0.013, P = 0.910; X2 = 2.443, P = 0.118) (Figure 3).

### 4.9. The Rate of HBV DNA < 300 copies/mL at Weeks 24 as a Predictive Factor of Efficacy During Treatment of Week 52

**Figure 4:**
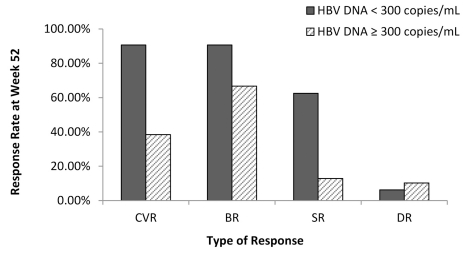
The Rate of HBV DnA < 300 Copies/mL at Weeks 24 as predictive actor During Treatment for efficacy of Week 52 . CVR, complete virological response; BR, biochemical response; SR, serological response

For 32 patients with HBV DNA < 300 copies/mL at Week 24, the CVR, BR, SR, and DR at week 52 were 90.63% (29/32), 90.63% (29/32), 62.50% (20/32), and 6.25% (2/32), respectively. But, for the other 39 patients with HBV DNA ≥ 300 copies/mL at Week 24, the CVR, BR, SR, and DR at Week 52 were 38.46% (15/39), 66.67% (26/39), 12.82% (5/39), and 10.26% (4/39), respectively. The differences in CVR, BR, and SR between the two groups were statistically significant (X2 = 20.295, P = 0.000; X2 = 5.780, P = 0.0016; X2 = 19.016, P = 0.000). But, the difference in DR between the two groups was not statistically significant (X2 = 0.365, P = 0.546) (Figure 4).

## 5. Discussion

The importance of optimizing therapy with nucleoside analogs lies in their long-term treatment course and inevitable resistance [3]. How to screen patients who are the most qualified targets for certain drugs is widely debated. During therapy, efficacy predictions are usually made to achieve the best effect, the lowest occurrence of resistance, and the highest effect:cost ratio. In recent years, optimization therapy with nucleoside analogs has focused on the baseline situation, considering early response and the application of roadmaps [4]. Due to its potent antiviral effect [5], relatively higher seroconversion rate of HBeAg and HbeAb [6], and affordable price, LdT is still widely used as the first option in naive CHB patients, especially for HBeAg-positive patients in certain countries, including China and other countries of Asia. Therefore, research on the predictive factors of antiviral efficacy of LdT is still important and can provide us with better evidence for optimization therapy. In this study, 71 CHB patients who received at least 52 weeks of LdT treatment were included. The CVR, BR, and SR were 61.97%, 77.46%, and 35.21% after 52 weeks of treatment, respectively. At the same time, the DR was 8.45% at Week 52. The type of gene mutation was mainly rtM204I. Another type was the rtLl80M + rtM204V alliance mutation. These results are consistent with other published data [7][8].

This study investigated several baseline host and viral factors that may influence antiviral efficacy, including age, gender, and baseline HBV DNA and ALT levels. The multiple regression statistical analyses showed that patient age and gender had no impact on CVR, BR, SR, or DR. Baseline ALT level significantly affected CVR, suggesting that higher ALT levels lead to a higher CVR, similar to results of the GLOBE clinical trial [9] and other reports [10][11]. Some scholars have proposed that better effects of LdT could be obtained in HBeAg-positive CHB patients with 10 to 20 times the upper limit of normal [12]. Further, the occurrence of SR correlates significantly with baseline HBV DNA and ALT levels, suggesting that patients with higher ALT and lower HBV DNA levels at baseline achieve E-antigen disappearance or seroconversion more easily, consistent with other reports11. At the same time, no baseline factor had an impact on BR or DR, perhaps due to too few cases with resistance for DR, unsuitable for statistical analysis. We tried to identify HBeAg-positive CHB patients who were most appropriate for Ldt treatment at baseline. ALT values and HBV DNA were analyzed and compared. The differences in CVR, SR, and DR between patients with baseline ALT > 120 IU/mL and ALT ≤ 120 Iu/ml were statistically significant, and the differences in SR between patients with baseline HBV DNA level > 107 copies/mL and ≤ 107 copies/mL were statistically significant, suggesting that patients with ALT > 120 and HBV DNA ≤ 107 copies/mL at baseline are more suitable for LdT therapy, similar to another report [13].

The optimization strategy also includes medication adjustments according to early response during treatment, except according to baseline factor [14]. For different drugs, alterations should be made at different time points due differing characteristics [15]. The roadmap concept was proposed according to the results of the Ldt GLOBE trial [8]. It was confirmed that patients with undetectable serum HBV DNA levels after 24 weeks had the best long-term outcomes, while those with levels remaining above 10,000 copies per ml were unlikely to benefit from long-term therapy [16]. In this study, the results showed that the differences in CVR, BR, and SR at 52 weeks was statistically significant between patients with HBV DNA < 300 copies/mL at Week 24 and those HBV DNA ≥ 300 copies/mL. HBV DNA < 300 copies/mL at Week 24 had good predictive value for CVR, BR, and SR at Week 52. A longer-term study was performed to show that Week 12 HBV DNA < 200 IU/mL was predictive of a greater chance of HBV DNA undetectability and a lower chance of resistance by Year 3. Undetectable HBV DNA at Week 24 was predictive of viral suppression at Year 2 but not at Year 3 [17]. The observation period of this study lasted only 1 year, and it is worth examining in an extended treatment course.

Of the 71 cases in this study, drug resistance occurred in 6 patients (8.45%). However, the observation period lasted only 52 weeks. With an extension of the treatment course, the occurrence of drug resistance may increase gradually. Therefore, it is necessary to monitor the occurrence of drug resistance with ongoing treatment. Usually, Ldt is regarded as a safe medicine, even for pregnant woman [18]. In this study, good safety was also confirmed. The adverse effects rate was only 5.63%, which were reversible, and it was not necessary to interrupt antiviral therapy. The rate was lower than reported [19]. In this study, virological effect, biochemical effect, and serological effects were used to value clinical efficacy. Histology remains the most exact and reliable index to value antiviral effects, but unfortunately, histological information and data were unavailable for our patients. This is a limitation of our study.

In summary, our results suggest the following conclusions: LdT has good efficacy in naïve HBeAg-positive chronic hepatitis B patients, with a good safety profile. In particular, there were more responders among patients with lower HBV DNA levels, especially less than 107 copies/ml, and higher ALT values, especially higher than 120 Iu/ml at baseline, to LdT therapy. At the same time, it is crucial to focus on the response at Week 24 during the course of treatment. If HBV DNA level is still > 300 copies/mL at Week 24, alterations or adjustments to the treatment strategy should be considered. The primary limitations of this study are the relatively short observation time period and the small number of patients. However, the results of this study suggest some clues and indicators of clinical value in the 52-week observation period. It is worth performing further studies by continuing treatment for longer terms.
